# Anti-RSV drug screening and inhibition of RSV infection by lapatinib through the IL-17 pathway

**DOI:** 10.1128/aac.01972-25

**Published:** 2026-06-25

**Authors:** Zheng Sun, Yixuan Huang, Rui Guo, Tao Jiang, Qingru Chen, Xinyu Zhang, Jiling Liu, Qi Tang, Mingwei Chen, Shenghai Huang

**Affiliations:** 1 School of Life Sciences, Anhui Medical University 428675 , Hefei, Anhui, China; 2 Department of Endocrinology, the First Affiliated Hospital of Anhui Medical University 12485, Hefei, Anhui, China; 3 Department of Microbiology, School of Basic Medical Sciences, Anhui Medical University 659392, Hefei, Anhui, China; 4 Department of Neurosurgery, Anhui Public Health Clinical Center (North District, the First Affiliated Hospital of Anhui Medical University) 12485, Hefei, Anhui, China; 5 The Institute of Clinical Virology, Anhui Medical University 12485, Hefei, Anhui, China; Chinese Academy of Medical Sciences & Peking Union Medical College, Beijing, China

**Keywords:** RSV, lapatinib, antiviral activity, inflammatory factors, IL-17

## Abstract

Respiratory syncytial virus (RSV) is a leading cause of severe lower respiratory tract infections in infants and young children worldwide. Safe and effective treatments for RSV remain limited. In this investigation, we screened more than 100 anti-influenza compounds from a small-molecule library using an A549 cell model. Among these drugs, lapatinib emerged as a promising candidate and demonstrated potent anti-RSV activity along with low cytotoxicity. Lapatinib not only significantly suppressed RSV replication *in vitro* but also markedly reduced viral proliferation and mitigated inflammatory lung injury in a BALB/c female mouse model. Mechanistic studies revealed that lapatinib inhibited RSV replication through the downregulation of interleukin-17 (IL-17). This effect was antagonized upon IL-17 overexpression, confirming the pivotal role of this cytokine. We further proposed that lapatinib exerted its antiviral effect by targeting ErbB1 and ErbB2 receptors, thereby disrupting IL-17–mediated viral propagation. These findings provide both novel mechanistic insights and a compelling therapeutic direction for the treatment of RSV infection。.

## INTRODUCTION

Respiratory syncytial virus (RSV) is a highly contagious RNA virus belonging to the *Pneumoviridae* family that is transmitted primarily through respiratory droplets and direct contact (1). It represents a leading cause of severe lower respiratory tract infections, such as bronchiolitis and pneumonia, in infants and young children (2). Epidemiological studies have indicated that nearly all children are infected with RSV by the age of two, with those under one year of age facing the highest risk of severe disease. During peak seasons, RSV accounts for more than 80% of acute respiratory infections in infants (3, 4). Beyond acute illness, RSV infection is associated with the development and exacerbation of reactive airway disease and asthma in pediatric populations (5, 6).

Despite the significant health burden associated with this disease, therapeutic options for treating RSV remain limited (7, 8). Ribavirin is the only nucleoside analog that is approved for treatment (9), yet its use is limited by modest efficacy, potential teratogenicity, and unclear mechanisms of action (10). Recent advances in prevention include the approval of long-acting monoclonal antibodies such as nirsevimab, which offers passive immunity for up to five months—covering a typical RSV season (11, 12)—for infants and high-risk young children (13). Additionally, vaccines have been approved for older adults (e.g., Arexvy, Abrysvo, and mRNA-1345) (14–17). However, direct immunization vaccines for the infant population and highly effective antiviral treatments remain core challenges that are yet to be urgently addressed in this field.

Given the prolonged timelines and high costs of *de novo* drug development, drug repurposing has emerged as a promising strategy for identifying new therapeutic applications for existing agents (18, 19), as exemplified by the transition of aspirin from an analgesic to an antiplatelet and chemopreventive agent (20, 21). Evidence suggests that a significant proportion of approved drugs may be suitable for such retargeting (22). Herein, we investigated lapatinib—a tyrosine kinase inhibitor targeting EGFR/ErbB1 and HER2/ErbB2 in breast cancer therapy (23)—which also exhibits antiviral activity against SARS-CoV-2 (24–26). The dual mechanisms of lapatinib arise from ErbB receptor inhibition: antitumor effects manifest through the blockade of ErbB autophosphorylation, leading to suppression of the MAPK/PI3K pathway and resulting in inhibition of proliferation with concurrent apoptosis induction (27), whereas antiviral effects occur when viral entry/replication (RSV-F protein binding to EGFR (28) requires ErbB signaling), where lapatinib blocks viral internalization and consequently downregulates inflammatory responses. Thus, the ErbB pathway serves as a convergent hub, enabling both tumor proliferation and viral hijacking of the host machinery. However, the efficacy of lapatinib against other respiratory viruses, including RSV, remains largely unexplored.

Through systematic drug screening, lapatinib was identified as a candidate with a high selectivity index. This study aims to evaluate the anti-RSV activity of lapatinib both *in vitro* and *in vivo* and to elucidate its mechanisms of action, with the goal of expanding the therapeutic arsenal against RSV and improving clinical outcomes.

## RESULTS

### Lapatinib has weaker cytotoxicity and better anti-RSV activity

**Fig 1:**
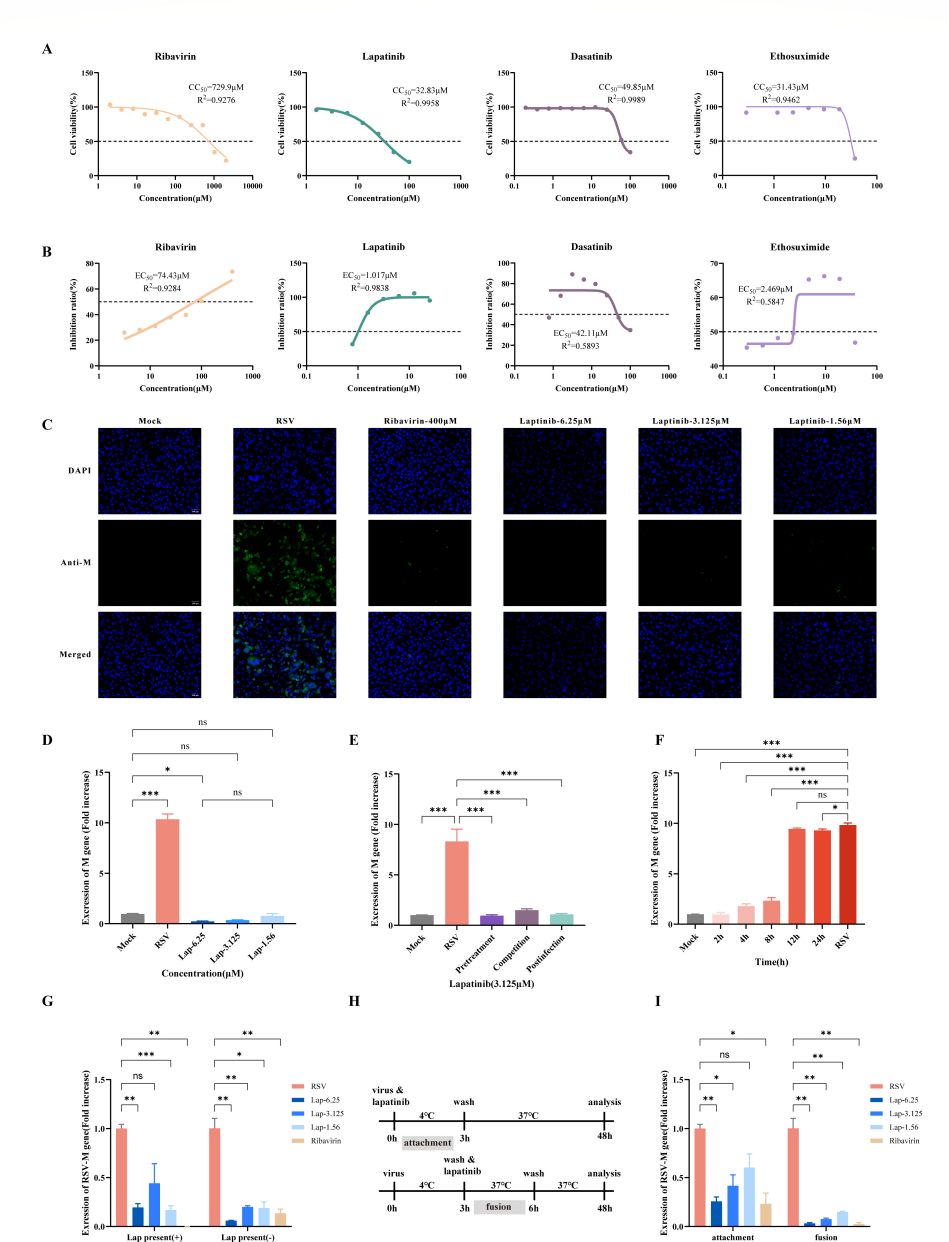
Anti-RSV drug screening and the antiviral effect of lapatinib. (**A**) Cytotoxicity of ribavirin, lapatinib, dasatinib, and ethosuximide at various concentrations (CC₅₀ values were determined by the CCK-8 assay). (**B**) Anti-RSV activity of the compounds at different concentrations (EC₅₀ values were calculated from CCK-8 data). (**C**) Immunofluorescence staining of RSV M protein expression in A549 cells. From left to right: mock (uninfected control), RSV-infected, ribavirin-treated, and lapatinib-treated groups (increasing concentrations). Scale bar = 50 µm. (**D**) Antiviral effects of lapatinib at graded concentrations. (**E**) Anti-RSV effects of lapatinib administered at different infection phases: preinfection, coinfection (competition), and postinfection. (**F**) Effects of lapatinib on RSV replication when it was added at different time points postinfection. (**G**) Effects of persistent and nonexistent lapatinib on RSV replication. Before infection with RSV, the compounds were either removed (−) or left on the cells (+). (**H**) Schematic illustration of the experimental procedure for temperature-shift experiments. (**I**) Analysis of virus replication status following the temperature-shift experiments using RT‒qPCR. The values shown are the averages of two independent experiments, each performed in triplicate ± standard deviation. The treatments included ribavirin (400 μM) and lapatinib: lap-6 (6.25 μM), lap-3 (3.125 μM), and lap-1 (1.56 μM). The data are presented as the means ± SDs. *P < 0.05, **P < 0.01, ***P < 0.001.

To identify new anti-RSV therapeutics, we employed a preexisting library of nearly 100 clinical drugs that were originally characterized for their anti-influenza virus activities. In this screen, A549 cells were coincubated with serial drug dilutions for 48 h, and cell viability was determined using a CCK-8 assay. Three drugs—ethosuximide, lapatinib, and dasatinib—were selected because of their low cytotoxicity and protective effects on RSV-infected cells. Subsequent evaluation compared the cytotoxicity and antiviral activity of these three candidates against ribavirin, an approved therapeutic for RSV infection used as a positive control. The results indicated that compared with ribavirin, lapatinib had a more potent antiviral effect (Fig. 1A and B). Specifically, lapatinib demonstrated a CC_50_ of 32.83 μM, an EC_50_ of 1.017 μM, and the highest selectivity index (SI = CC_50_/EC_50_) of 32.48 among all the tested drugs, including ribavirin. Therefore, we propose that the anti-RSV activity of lapatinib warrants further research. Since lapatinib was screened from an anti-influenza drug library, before studying its effect against RSV, we investigated the chemical structure of lapatinib and its antiviral activity (Fig. S1A and B).

### Lapatinib inhibits RSV replication during early infection

We first evaluated the dose-dependent effect of lapatinib on RSV infection at noncytotoxic concentrations. We used immunofluorescence to detect the RSV M protein to verify the effects of different concentrations of the drug on viral protein expression after treatment. The results revealed that the inhibitory effect of low-dose lapatinib was comparable to that of 400 μM ribavirin (the concentration is typically lower than its IC₅₀) (Fig. 1C). Moreover, RT‒qPCR analysis revealed that lapatinib significantly inhibited RSV replication in a concentration-dependent manner. The inhibitory effect remained stable between 3.125 μM and 6.25 μM, with no significant difference between the two concentrations (Fig. 1D). On the basis of its cytotoxicity profile, a concentration of 3.125 μM lapatinib was selected for the subsequent experiments. To investigate the stage of the viral life cycle that is targeted by the compound, it was coincubated with RSV-infected cells starting at three different time points: 2 h before infection, at the time of infection, or 2 h after infection. Compared with the control treatment, the lapatinib treatment significantly suppressed RSV M gene expressions at all three time points (Fig. 1E), suggesting that it may inhibit viral adsorption, entry, and replication. To further elucidate the effective window of inhibition, a time-of-addition assay was performed in which the drug was added at various times postinfection, and viral inhibition was measured at 48 h. Lapatinib exhibited a potent inhibitory effect on RSV replication only when it was added within the first 8 h postinfection (Fig. 1F), indicating that it primarily acts during the early stages of the RSV life cycle. To verify whether the antiviral effect of lapatinib is mediated by blocking virus absorption and entry into cells, we altered drug exposure conditions using a drug washout assay to assess its impact on RSV replication. Results demonstrated that continuous drug presence significantly enhanced RSV inhibition (Fig. 1G). Subsequent temperature-shift experiments (Fig. 1H) (29) further evaluated lapatinib’s effect on RSV adsorption and fusion/entry processes. These data confirm that lapatinib predominantly targets RSV absorption and cellular entry, with a more pronounced ability to prevent virus-cell fusion (Fig. 1I).

### Lapatinib inhibits the expression of multiple cytokines after RSV infection

**Fig 2:**
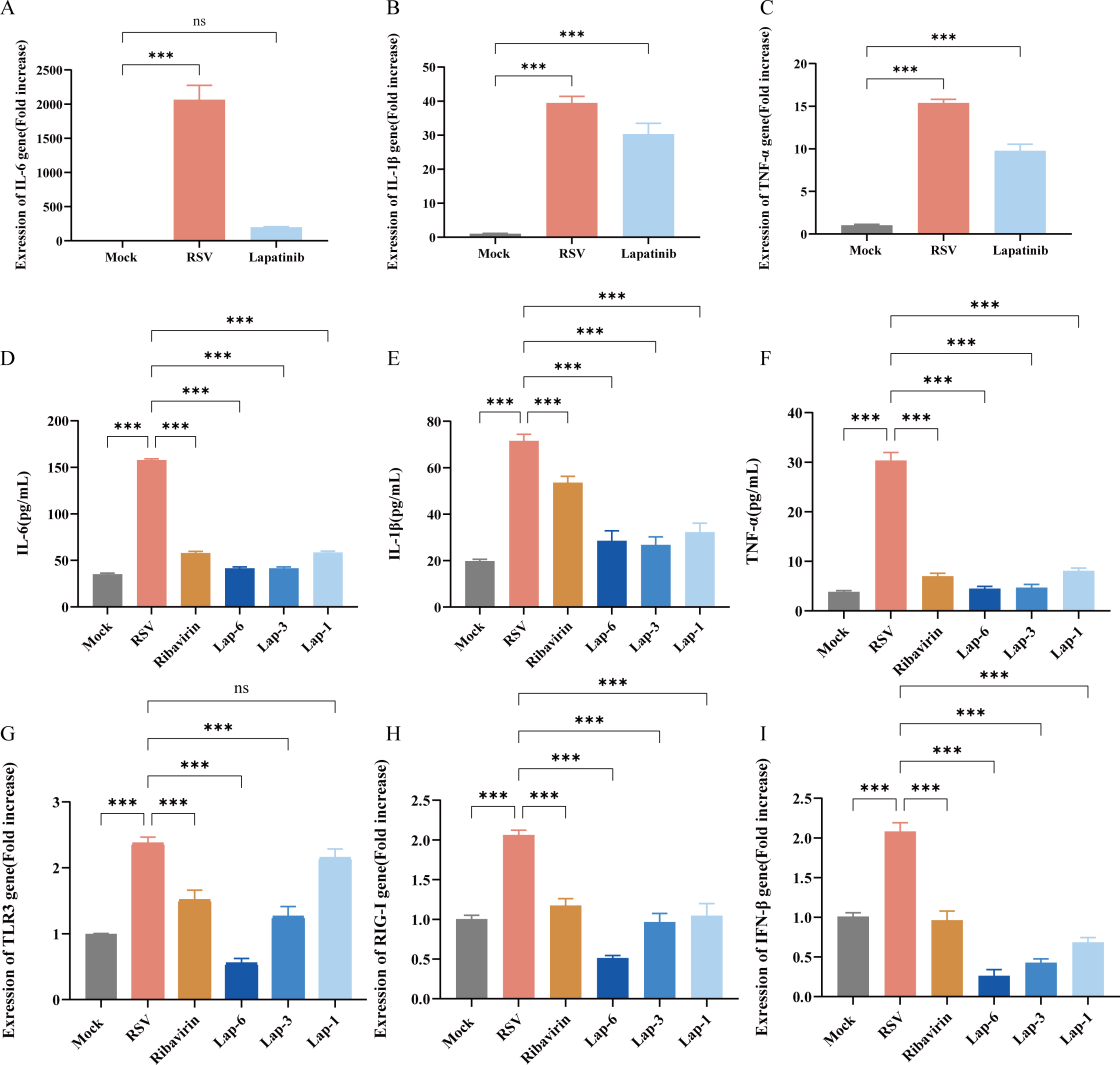
Effect of drugs on inflammatory factors in RSV-infected cells. (**A–C**) mRNA expression of IL-6, IL-1β, and TNF-α in A549 cells; the drug concentration of lapatinib was 3.125 μM. (**D–F**) mRNA expression of TLR-3, RIG-I, and IFN-β in A549 cells. (**G–I**) Protein levels of IL-6, IL-1β, and TNF-α in A549 cell culture supernatant, as determined by ELISA. The treatments included ribavirin (400 μM) and lapatinib: lap-6 (6.25 μM), lap-3 (3.125 μM), and lap-1 (1.56 μM). The data are presented as the means ± SDs. *P < 0.05, **P < 0.01, ***P < 0.0001.

We next investigated the effect of lapatinib on the virus-induced inflammatory response. RSV infection significantly upregulated the expression of the proinflammatory cytokines IL-6, IL-1β, and TNF-α. Lapatinib treatment markedly suppressed these upregulations, with a particularly pronounced inhibition of IL-6 (Fig. 2A through C). Consistent with the mRNA data, enzyme-linked immunosorbent assays (ELISAs) of cell culture supernatants confirmed that lapatinib potently reduced the secretion of these cytokines. Notably, compared with ribavirin, lapatinib at a low dose had superior inhibitory effects on IL-6, IL-1β, and TNF-α production (Fig. 2D through F), suggesting that it is a more potent suppressor of RSV-induced inflammation at lower concentrations.

We further explored the potential mechanism by examining pattern recognition receptors (PRRs), which are critical for initiating innate immune responses against viral infections (30). The TLR-3 and RIG-I signaling pathways are known to be pivotal in antiviral immunity. Our results revealed that both lapatinib and ribavirin inhibited the RSV-induced upregulation of TLR-3 and RIG-I expression. However, the inhibitory effects of ribavirin on TLR-3, RIG-I, and the downstream effector IFN-β were significantly weaker than those of lapatinib, despite the latter being used at a lower dose (Fig. 2G through I).

Collectively, these findings suggest that the antiviral activity of lapatinib is associated with the suppression of key proinflammatory cytokines and the modulation of critical PRR signaling pathways.

### Lapatinib ameliorates lung injury and the expression of inflammatory factors after RSV infection

**Fig 3:**
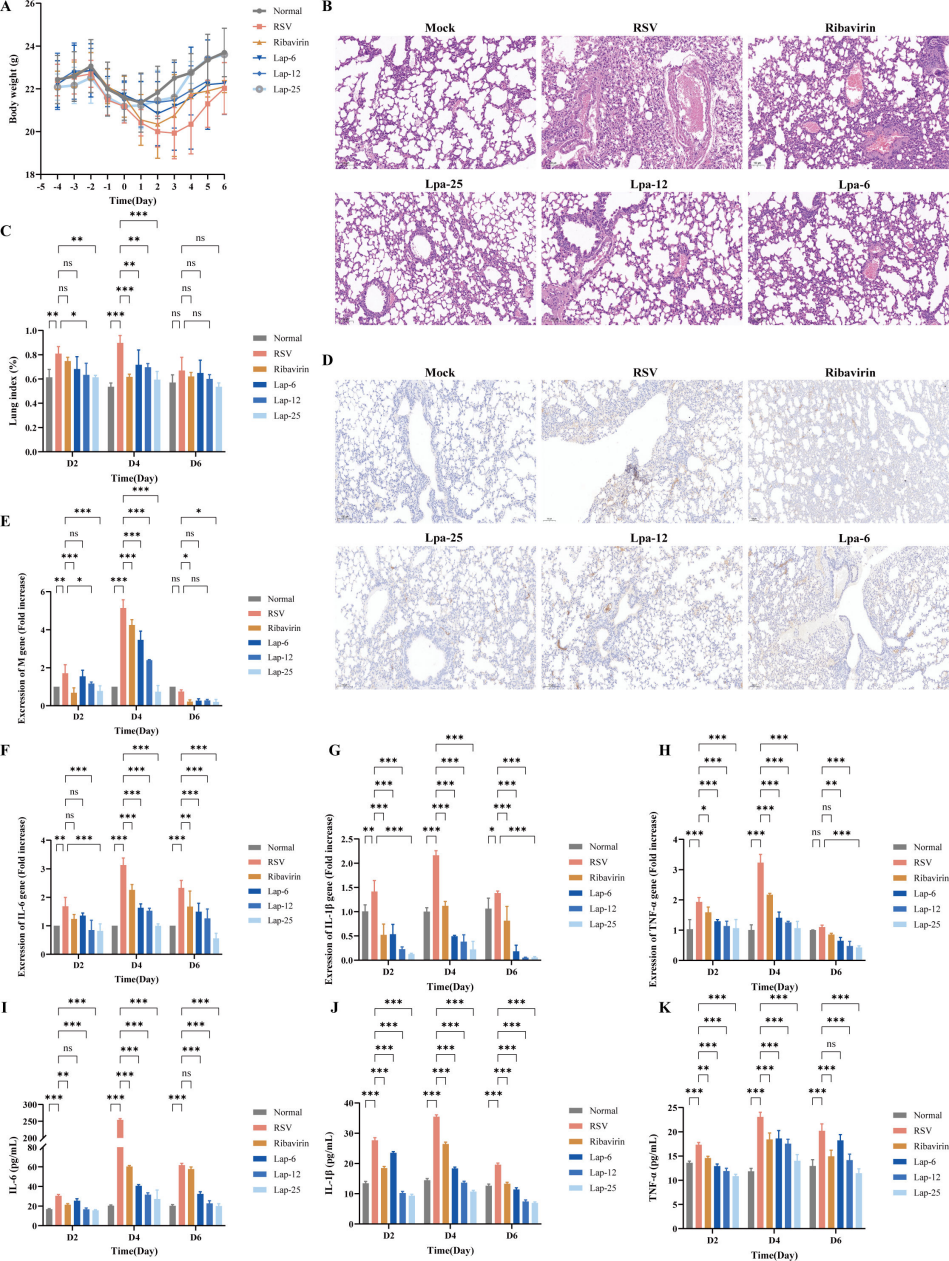
Anti-RSV activity of lapatinib in mice. (**A**) Changes in body weight of BALB/c mice infected with RSV. (**B**) H&E staining of lung tissue on day 4 postinfection (scale bar = 100 µm). (**C**) Changes in the lung index of mice 6 days after infection with RSV; lung index = mouse lung weight/mouse body weight × 100%. (**D**) IHC staining of mouse lung tissue on day 4 of RSV infection (scale bar = 100 µm). (**E**) Viral contents of mouse lung tissues. (**F–H**) mRNA expressions of the proinflammatory factors IL-6, IL-1β, and TNF-α in lung tissues. (**I–K**) Expression levels of IL-6, IL-1β, and IFN-β in mouse lungs over the 6-day treatment period. The mice were infected intranasally with RSV on day −1 (twice total), and drug treatment was initiated on day 0. The treatment groups received ribavirin (50 mg/kg/d), lapatinib at high (Lap-25: 25 mg/kg/d), medium (Lap-12: 12.5 mg/kg/d), or low (Lap-6: 6.25 mg/kg/d) doses. The data are presented as the means ± SDs. *P < 0.05, **P < 0.01, ***P < 0.001.

To evaluate the therapeutic efficacy of lapatinib *in vivo*, we established a mouse model of RSV infection. Mice were infected intranasally with RSV and subsequently treated via intragastric administration of lapatinib or ribavirin, with a control group receiving normal saline. Daily monitoring revealed that mice in the lapatinib group exhibited greater recovery of body weight gain than those in the ribavirin- and RSV-infected groups did (Fig. 3A), with no significant differences observed in general behavior.

We employed the lung index as a key metric to assess drug efficacy, where a higher index indicates more severe pulmonary pathology. RSV infection induced lung inflammation, leading to increased lung weights. Lapatinib treatment significantly reduced the lung index, demonstrating its ability to alleviate virus-induced lung injury. Notably, at a higher dose, lapatinib was more effective than ribavirin (Fig. 3C). Histopathological examination of lung tissues via H&E staining revealed that lapatinib markedly improved RSV-induced pathological changes, including inflammatory cell infiltration and alveolar septum thickening. This protective effect was dose-dependent and became more pronounced with increasing drug concentrations (Fig. 3B). Furthermore, immunohistochemical (IHC) staining revealed that both ribavirin and lapatinib inhibited viral antigen expression in infected lungs to varying degrees (Fig. 3D). Consistent with these findings, RT‒qPCR analysis of lung tissues confirmed that lapatinib potently suppressed the expression of the RSV M gene at its peak (day 4 postinfection), indicating strong antiviral activity (Fig. 3E).

Analysis of inflammatory mediators in lung tissue revealed that lapatinib significantly downregulated the expressions of IL-6, IL-1β, and TNF-α at 2, 4, and 6 days postinfection, outperforming ribavirin (Fig. 3F through H). This anti-inflammatory profile was corroborated by ELISA measurements of serum cytokine levels, which peaked on day 4 after infection but subsequently decreased. Compared with ribavirin, lapatinib more strongly inhibited these serum cytokines despite being administered at a lower dose (Fig. 3I through K).

These collective results underscore the therapeutic potential of lapatinib in mitigating RSV-associated lung inflammation and viral replication, identifying it as a promising candidate for further development.

### The IL-17 signaling pathway may be a target for drug therapy

**Fig 4:**
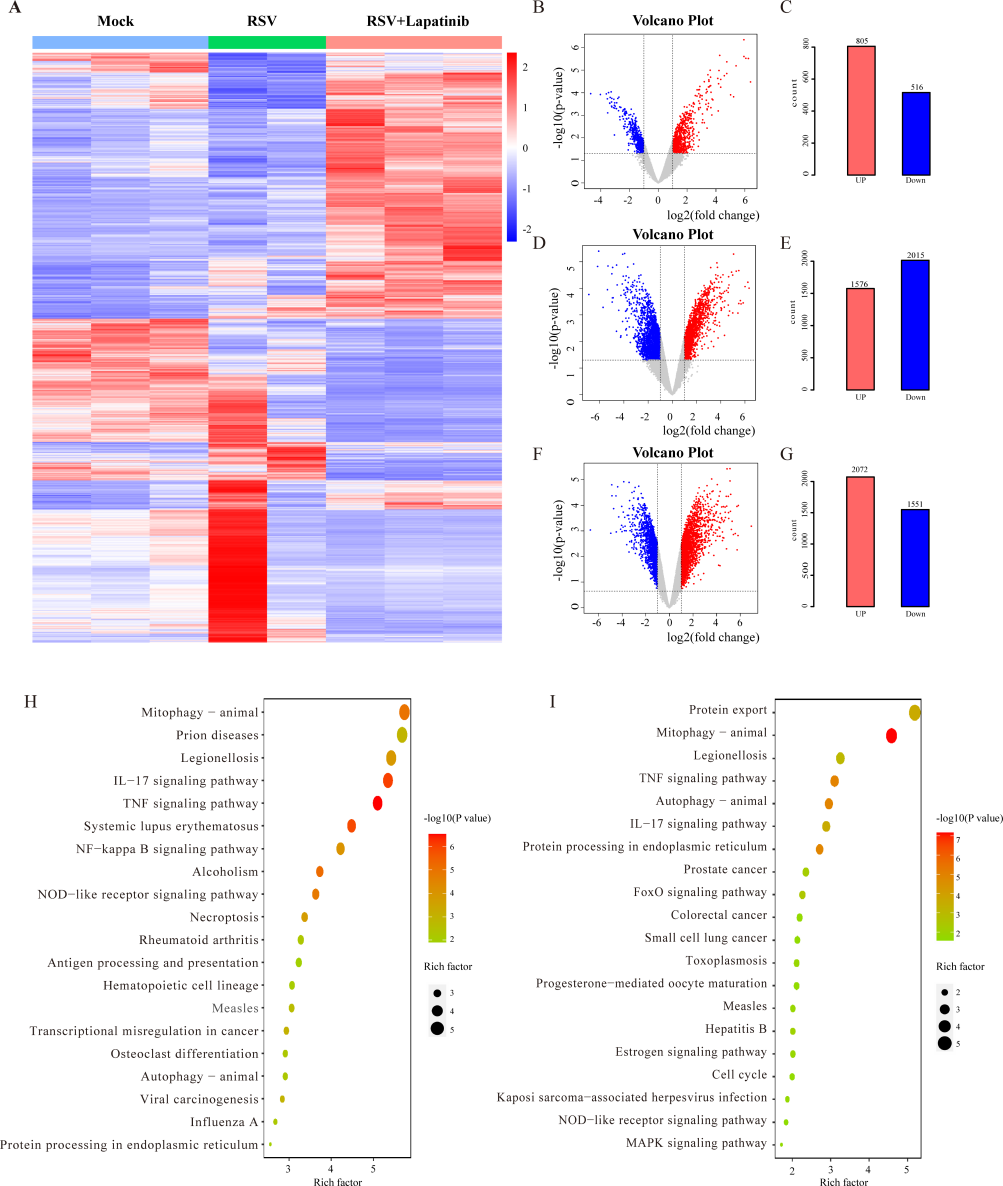
Differential gene enrichment analysis. (**A**) Heatmap (DEGs) of differentially expressed genes with a lapatinib drug concentration of 3.125 μM. (**B, D, & F**) Volcano plots of DEGs for the comparisons of RSV vs. mock, lapatinib vs. RSV and lapatinib vs. mock, respectively. Red and blue dots represent significantly up- and downregulated genes, respectively. (**C, E, & G**) Bar plots summarizing the numbers of upregulated (red) and downregulated (blue) DEGs for each comparison. (**H**) KEGG pathway enrichment analysis of upregulated genes in the RSV vs. mock group. (**I**) Enrichment analysis of downregulated genes in the lapatinib vs. RSV group.

To identify the potential target of lapatinib in the treatment of RSV infection, we performed whole-genome sequencing and bioinformatics analyses to construct heatmaps of the differentially expressed genes (DEGs) across the mock group, the RSV-infected group, and the group treated with 3.125 μM lapatinib after RSV infection (Fig. 4A). The expression patterns of these DEGs were further visualized using volcano plots and histograms (Fig. 4B through G).

GO functional enrichment analysis of the DEGs revealed that the genes whose expression significantly changed were associated with a broad spectrum of biological functions (Fig. S2A through C). Subsequently, the Kyoto Encyclopedia of Genes and Genomes (KEGG) database was used as the reference resource. Significantly enriched pathways were defined as those with an adjusted *P*-value (FDR) < 0.05 according to the Benjamini–Hochberg correction. The results show that following RSV infection, the upregulated genes were enriched primarily in the IL-17 signaling pathway, TNF signaling pathway, and NF-κB signaling pathway (Fig. 4H), whereas no significant changes were observed in the downregulated genes (Fig. S3A). In contrast, in the lapatinib-treated group, the IL-17 signaling pathway was identified among the pathways enriched for downregulated genes (Fig. 4I), leading us to hypothesize that IL-17 may be a target of lapatinib in this context. Moreover, the upregulated genes in both the lapatinib-treated group and the mock control group were enriched mainly in the mitophagy pathway (Fig. S3B through D). We propose that this may be linked to RSV infectivity and the inhibitory effect of lapatinib, given that mitophagy can play dual roles in RSV infection.

### Lapatinib significantly inhibited the expression of the IL-17 gene

**Fig 5:**
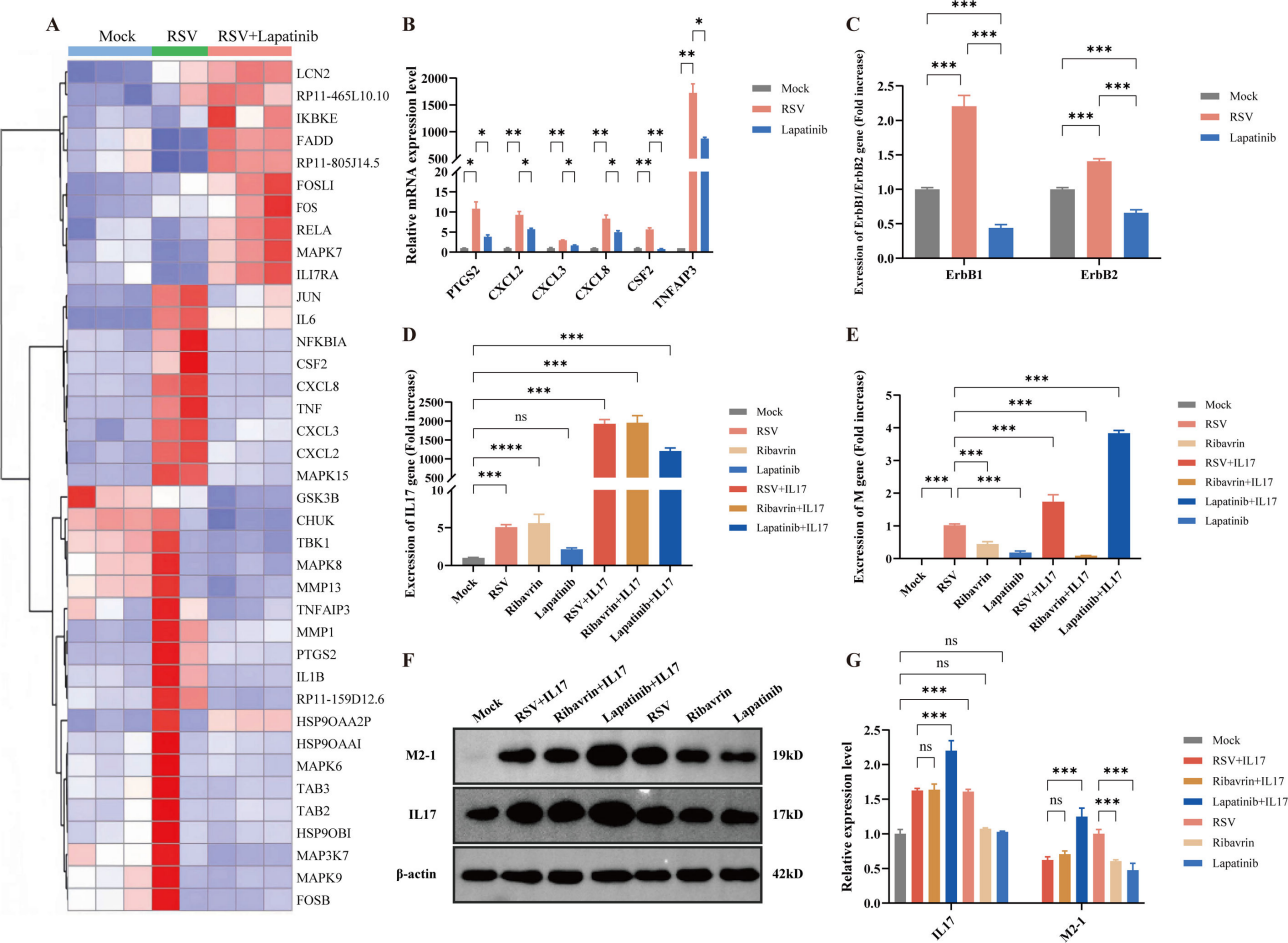
IL-17 signaling pathway-related genes and detection of lapatinib targets of action. (**A**) Heatmap of genes involved in the IL-17 signaling pathway. (**B**) Expression of representative genes of the IL-17 signaling pathway. (**C**) Gene expression of ErbB1 and ErbB2. (**D**) Changes in IL-17A expression after RSV infection and drug treatment. (**E**) Overexpression of IL-17A and its effect on viral replication after drug treatment. (**F**) Differences in the molecular expression of the RSV M2-1 and IL-17 proteins after drug treatment. (**G**) Gray value analysis results of F by ImageJ. Drug concentrations: 400 μM ribavirin; 3.125 μM lapatinib. Data represent the means ± SDs. *P < 0.05, **P < 0.01, ***P < 0.001.

On the basis of predictions from the sequencing results, we first mapped the up- and downregulated genes associated with the IL-17 signaling pathway, and multiple genes within this pathway were downregulated following lapatinib treatment (Fig. 5A). We then selected representative IL-17 signaling pathway genes—PTGS2, CXCL2, CXCL3, CXCL8, CSF2, and NF AIP3—for validation. The results confirmed that their expression was significantly reduced after drug treatment (Fig. 5B).

ErbBs are known to mediate inflammation and lung injury and act as key regulators of inflammatory processes that contribute to barrier dysfunction, thrombosis, vasoconstriction, and subsequent fibrosis in epithelial cells (31–33). Lapatinib has been reported to target both ErbB1 and ErbB2 (34), and RSV has been shown to induce ErbB1 activation (35). In our study, lapatinib markedly suppressed the expression of ErbB1 and ErbB2 after RSV infection (Fig. 5C), suggesting that lapatinib may exert its effects by acting on these two targets, thereby inhibiting viral replication and inflammatory factor expression.

We next treated IL-17A-overexpressing cells with lapatinib and observed that it antagonized IL-17A expression (Fig. 5D). Furthermore, measurement of viral nucleic acid levels in IL-17A-overexpressing cells revealed that the ability of lapatinib to inhibit viral replication was abolished, whereas the antiviral effect of the control drug ribavirin remained unaffected (Fig. 5E). Under the same conditions, protein-level analysis of IL-17A and RSV M2-1 yielded results consistent with those at the gene level (Fig. 5F and G). Taken together, these findings indicate that lapatinib inhibits RSV replication by suppressing IL-17 expression and that its antiviral activity is antagonized when IL-17 is overexpressed.

## DISCUSSION

At present, established RSV prevention and treatment strategies, such as nirsevimab, palivizumab (a neutralizing antibody prophylactic for high-risk children), and ribavirin (a synthetic nucleotide analog with broad-spectrum antiviral properties), may not offer enhanced therapeutic benefits and primarily provide supportive rather than curative effects (7, 36, 37). When managing nonsevere RSV infections, conventional treatment strategies typically focus on alleviating symptoms similar to those of the common cold, including rest and maintaining adequate water intake to maintain fluid balance. However, current therapeutic drugs require intramuscular or intravenous injections, causing discomfort and sequelae in patients. Therefore, the development of new antiviral drugs, especially universal antiviral drugs, to effectively combat RSV is urgently needed.

This study identified lapatinib as a highly effective anti-RSV candidate from a clinical drug library containing more than 100 known anti-influenza agents. At the cellular level, compared with the conventional drug ribavirin, lapatinib demonstrated superior antiviral activity and a higher therapeutic index. Our findings indicate that lapatinib primarily exerts a prophylactic effect by blocking viral absorption and entry. It effectively suppressed viral replication during the early stages of infection (within 8 h postinfection). In a mouse model of RSV infection, treatment with lapatinib significantly reduced viral replication in lung tissue and alleviated pathological inflammatory damage. The *in vivo* efficacy of lapatinib was also superior to that of ribavirin.

The core innovation of this study lies in the systematic exploration of the potential of drug repositioning in antiviral therapy, with a particular focus on the anti-RSV mechanism of the anticancer drug lapatinib. We revealed that lapatinib can significantly alleviate the cytopathic effects and pulmonary inflammatory damage caused by RSV by specifically inhibiting the IL-17 signaling pathway and reducing the expression of key inflammatory cytokines (such as IL-6 and TNF-α). We speculate that RSV infection activates the ErbB pathway, which in turn regulates the expression of IL-17A/F-related genes (such as CXCL1 and MMP9) in the NF-κB/MAPK pathway (38, 39). Lapatinib inhibits ErbB signaling, blocks the activation of NF-κB/MAPK, and ultimately suppresses the expression of IL-17 (40). NF-κB, as an intermediate mediator, promotes the expression of IL-17, while IL-17 activates downstream pathways (such as Act1-TRAF6-TAK1) through IL-17R, further activating NF-κB and forming an “IL-17/NF-κB positive feedback loop,” and this core mechanism plays a role in many chronic diseases (such as chronic obstructive pulmonary disease and kidney injury) (41). Our experiments verified that the overexpression of IL-17 can reverse the antiviral effect of lapatinib, consolidating the crucial role of the IL-17 pathway and providing direct evidence for targeted therapy.

Compared with current antiviral drug development strategies, which predominantly focus on directly targeting viral proteins, this study takes a different approach by targeting host immune response modulation, offering a new perspective for addressing viral drug resistance. Research on the activity of lapatinib against other viruses, such as SARS-CoV-2, has revealed its unique ability to inhibit key host kinases that are essential for viral replication. Our study is the first to clearly elucidate the unique mechanism through which lapatinib acts against RSV via the IL-17 pathway. This discovery not only distinguishes it from its potential mechanism against coronaviruses but also highlights its unique advantage in treating RSV infection—simultaneously inhibiting viral replication and mitigating excessive inflammatory responses, achieving a “two-pronged” therapeutic effect.

Currently, research on antiviral agents has entered a new phase (42, 43). Multiple research teams are employing various methods, such as high-throughput screening, organoid infection models, and preclinical animal experiments, to systematically evaluate their antiviral spectra and mechanisms of action (44). By integrating multilevel evidence from cell models, gene overexpression, and signaling pathway analysis, this study establishes a complete chain of evidence from molecular mechanisms to phenotypic validation, providing a solid theoretical foundation for advancing the clinical translation of lapatinib. These findings not only offer a new candidate drug for RSV treatment but also, more importantly, open new directions for the development of broad-spectrum antiviral strategies targeting host factors. Future efforts to optimize the specificity and safety of IL-17 pathway inhibitors hold promise for the development of more targeted antiviral therapies.

## MATERIALS AND METHODS

### Cells, viruses, and drugs

HeLa cells were acquired from the ATCC cell bank. Human laryngeal epithelial cells (HEp-2) and human lung cancer cells (A549) were provided by the Department of Microbiology at Anhui Medical University. The RSV Long strain, also obtained from the same department, was propagated in HEp-2 cells, followed by purification and storage in liquid nitrogen. Viral titration was performed on HEp-2 cells using a plaque assay (45, 46), confirming a titer of 2.5 × 10^6^ PFU/mL. The small-molecule drug library used in this study, which contains nearly one hundred compounds, was sourced from Selleck, MedChemExpress, and Shanghai Taosu Biochemical Company (lapatinib source, purity 99.81%).

### Antibodies & reagents

A CCK-8 Kit was purchased from Beyotime Biotechnology (Shanghai, China), and a real-time PCR Kit was purchased from Monad (Wuhan, China). Human IFN-β enzyme-linked immunosorbent assay (ELISA) Kits, human IL-1β ELISA Kits, mouse IL-6 mini plate ELISA kits, mouse IL-1β mini plate ELISA Kits, and mouse IFN-β mini plate ELISA Kits were all purchased from Hefei Xishi Biological Co., Ltd.

### Laboratory animals

BALB/c mice (both female and male) were purchased from Zhejiang Weitong Lihua Laboratory Animal Technology Co., Ltd. (Certificate No. SCXK [Zhejiang] 2019–0001) and housed in the P2-level animal facility of the Animal Experiment Center at Anhui Medical University. For breeding, female and male mice were paired at a 2:1 ratio. From the offspring, 72 female BALB/c mice aged 6–8 weeks (approximately 22–24 g each) were selected for the experiments.

The mice were randomly assigned to six groups (*n* = 12 per group): normal control (mock), RSV infection, ribavirin positive control, and high-, medium-, and low-dose lapatinib groups. To establish infection, mice were intranasally inoculated twice with 50 μL of RSV viral suspension (the normal control group received serum-free DMEM). Drug treatments were administered as follows: the ribavirin group received 50 mg/kg/day, while the lapatinib groups received 25, 12.5, and 6.25 mg/kg/day. All treatments were given once daily at a dose of 200 μL, and the RSV infection and normal control groups received equivalent volumes of normal saline.

Throughout the treatment period post-RSV infection, the mice were observed and recorded daily. On days 2, 4, and 6 after lapatinib administration, four mice per group were randomly selected, and intact lung tissues were collected. The extent of the lung lesions was documented, and the lung weights were measured. The left lung was fixed for hematoxylin and eosin (H&E) staining and immunohistochemistry (IHC), while the right lung was flash-frozen in liquid nitrogen and stored at –80°C for subsequent RT‒qPCR analysis.

### Drug toxicity and antiviral activity assays

A549 cells in the logarithmic growth phase were seeded in 96-well plates at a density of approximately 10⁴ cells per well and cultured for 24 h. Following a 48-h coincubation with gradient concentrations of the compounds, 10 μL of CCK-8 solution was added to each well. The absorbance (A) was then measured, and the half-maximal cytotoxic concentration (CC_50_) was calculated. On the basis of the results of preliminary experiments, an MOI of five for the RSV Long strain was selected for drug screening, as this infection condition resulted in approximately 50% cell death in A549 cells after 48 h. For the screening assay, the culture medium was removed 2 h postinfection and replaced with medium containing gradient concentrations of the drugs. After 48 h of incubation, a mixture of CCK-8 reagent and maintenance medium (1:9) was added to each well, followed by further incubation for 30 min to 1 h. The absorbance was measured, and the half-maximal effective concentration (EC_50_) was calculated.

### Wash assays and temperature-shift experiments

For the experiment, monolayer A549 cells in 24-well plates seeded the day before were used and maintained in DMEM medium supplemented with 1% FBS. For wash assays, monolayers were pretreated with 3.125 μM lapatinib (37°C, 1 h), washed 3× with PBS, then infected with virus in fresh virus-containing medium. Viral titers were quantified 48 h postinfection. For temperature-shift-based attachment analysis, cells were pre-chilled (4°C, 1 h), coincubated with serially diluted lapatinib & virus (4°C, 3 h), washed 3× with PBS after inoculum removal, and incubated in fresh medium (37°C, 5% CO_2_, 48 h). Fusion analysis followed identical initial infection; after virus removal, lapatinib was added and cells were incubated (37°C, 3 h) to permit fusion before medium replacement and final incubation (37°C, 5% CO_2_, 48 h). Viral replication in all assays was assessed by RT‒qPCR at 48 h.

### Immunofluorescence detection of the RSV M protein

A549 cells in the logarithmic growth phase were seeded in 12-well plates and infected with RSV. Following infection, the cells were treated with various drugs for 48 h. After treatment, the medium was discarded, and the cells were fixed with 4% paraformaldehyde for 15–20 min. After fixation, the cells were washed several times with PBS and permeabilized with 0.2%–0.5% Triton X-100 for 15 min. After another PBS wash, the cells were blocked with 5% BSA for 30 min. Subsequently, the cells were incubated overnight at 4°C with a primary antibody against RSV M protein (diluted 1:200 in PBS, 200 μL per well). The next day, after being washed, the cells were incubated with a fluorescent secondary antibody (diluted 1:200 in PBS containing 0.5% BSA, 200 μL per well) for 1 h in the dark. After being washed again, the cells were incubated with diluted DAPI (1:200 in PBS with 0.5% BSA) for 10 min. Finally, the cells were washed 5 times with PBS and visualized under a fluorescence microscope.

### Overexpression of the IL-17A protein

The full-length coding sequence (CDS) of the human IL-17A gene (NM_002190.3) was cloned and inserted into the pcDNA3.1(+) expression vector to construct the IL-17A overexpression plasmid, which was synthesized by Nanjing Qingke Biotechnology Co., Ltd. A549 cells seeded in 6-well plates were transfected with the plasmid using Lipo8000 transfection reagent (Beyotime Biotechnology). Each well of the IL-17A overexpression group received 2.5 μg of the plasmid, while the other groups were transfected with an empty vector as a control, and the overexpression was verified at the mRNA and protein levels (Fig. S4A). At 24 h post-transfection, the cells were infected with 50 μL of RSV suspension per well, and the mock group received an equal volume of culture medium instead. Concurrently, the following drug treatments were administered: ribavirin at 400 μM and lapatinib at 3.125 μM. After 48 h of drug treatment, the protein expression levels of IL-17A and RSV were analyzed by RT‒qPCR and Western blotting.

### Real-time PCR assay (SYBR Green)

The transcriptional levels of target genes, including RSV-M, ErbB1, ErbB2, and IL-6, were assessed by real-time quantitative PCR (RT‒qPCR). GAPDH was used as the internal reference gene for data normalization, and the relative fold changes in gene expression were calculated using the 2^−ΔΔCt^ method. The sequences of all the primers used are provided in Table S1. The RT‒qPCR procedure was conducted under the following thermal cycling conditions: initial denaturation at 95°C for 10 min, followed by 40 cycles of denaturation at 95°C for 10 sec and annealing/extension at 60°C for 30 sec.

### Western blot analysis

The protein expression levels of RSV M2-1 and IL-17 were analyzed by Western blotting. The proteins were separated by 12% SDS‒PAGE and transferred to PVDF membranes. The membranes were then incubated overnight at 4°C with the following primary antibodies: anti-RSV M2-1, anti-IL-17 (1:1000 dilution), and anti-β-actin (1:2000 dilution). The band intensities were quantified using ImageJ software (National Institutes of Health, Bethesda, MD, USA), and the expression level of each target protein was expressed as the ratio of its grayscale value to that of β-actin.

### Statistical analysis

All the data were statistically analyzed using SPSS version 22.0; differences between groups were assessed by two-way ANOVA, and precise *P* values for both sides were reported. GraphPad Prism 8 was used for graphing. (*) *P* < 0.05 indicated a statistically significant difference, and (**) *P* < 0.01 indicated a significant difference.

## SUPPLEMENTAL MATERIAL

Supplemental material aac.01972-25-s0001.docx Table S1; Fig. S1 to S4.

ASM does not own the copyrights to Supplemental Material that may be linked to, or accessed through, an article. The authors have granted ASM a non-exclusive, world-wide license to publish the Supplemental Material files. Please contact the corresponding author directly for reuse.
