# Supplementary material for: Anti-RSV drug screening and inhibition of RSV infection by lapatinib through the IL-17 pathway
Source: Antimicrob Agents Chemother. 2026 Jun 25;70(8):e01972-25. doi: 10.1128/aac.01972-25 (PMC13436392; doi:10.1128/aac.01972-25)
Supplement: Supplementary file 1 [file aac.01972-25-s0001.docx]

**Anti-RSV drug screening and inhibition of RSV infection by lapatinib through the IL-17 pathway**

Zheng Sun^2†^, Yixuan Huang^3†^, Rui Guo^1^, Tao Jiang^4^, Xinyu Zhang^1^, Jiling Liu^1^, Qi Tang^1*^, Mingwei Chen^3*^, Shenghai Huang^1,2,4,5*^

^1^ Department of Microbiology, School of Basic Medical Sciences, Anhui Medical University, Hefei, 230032, Anhui Province, China.

^2^ School of Life Sciences, Anhui Medical University, Hefei, 230032, Anhui Province, China.

^3^ Department of Endocrinology, the First Affiliated Hospital of Anhui Medical University, Hefei, 230022, Anhui Province, China.

^4^ Department of Neurosurgery, Anhui Public Health Clinical Center (North District, the First Affiliated Hospital of Anhui Medical University), Hefei, 230012, Anhui Province, China.

^5^ The Institute of Clinical Virology, Anhui Medical University, Hefei, 230032, Anhui Province, China.

† These authors contributed equally to this work.

Correspondence: Shenghai Huang, shhuang@ahmu.edu.cn; Mingwei Chen, chmw1@163.com; Qi Tang, tangqi@ahmu.edu.cn.

**Supplementary Table**

**Table 1. Sequences of the primers used in the RT-qPCR assay**

| Target gene | Sequences (5’→3’) |
| --- | --- |
| Human  GAPDH  GAPDH  RSV-M  RSV-M  ErbB1  ErbB1  ErbB2  ErbB2  IL-1β  IL-1β  IL-6  IL-6  TNF-ɑ  TNF-ɑ  TLR-3  TLR-3  RIG-I  RIG-I  IFN-β  IFN-β  PTGS2  PTGS2  CXCL3  CXCL3  CXCL2  CXCL2  CXCL8  CXCL8  CSF2  CSF2  TNFAIP3  TNFAIP3  Mouse  GAPDH  GAPDH  IL-6  IL-6  IL-1β  IL-1β  TNF-ɑ  TNF-ɑ | F： 5’-CTGGGCTACACTGAGCACC-3’  R： 5’-AAGTGGTCGTTGAGGGCAATG-3’  F： 5’-AACTTCACGAAGGCTCCACATACAC-3’  R： 5’- TCTGCTGGCATGGATGATTGGAAC-3’  F： 5’- ACCCATATGTACCATCGATGTC-3’  R：5’- GAATTCGATGATCAACTCACGG-3’  F：5’- CCAGCTCTTTGAGGACAACTAT -3’  R：5’- TTTCAAGATCTCTGTGAGGCTT-3’  F：5’- CTGAACTCAACTGTGTGAAATGC-3’  R：5’- TGATGTGCTGCTGCGAGA-3’  F：5’- ACACATGTTCTCTGGGAAATCGT-3’  R：5’- AAGTGCATCATCGTTGTTCATA-3’  F：5’- ACTGGCAGAAGAGGCACTC-3’  R：5’- CTGGCACCACTAGTTGGTTG-3’  F：5’- CAACTTAGCACGGCTCTGGA -3’  R：5’- CTGAAAGCTGGCCCGAAAAC-3’  F：5’- CTGGTTCCGTGGCTTTTTGG-3’  R：5’- CACCTGCCATCATCCCCTTA-3’  F：5’- CGTGGGAGATGTCCTCAACT-3’  R：5’- AGATCTCTGCTCGGACCACC-3’  F：5’- AGGAGGTCTTTGGTCTGGTG-3’  R：5’- TAGCCTGCTTGTCTGGAACA-3’  F：5’- AACAAGGGGAGCACCAACTG-3’  R：5’- ACCCTGCAGGAAGTGTCAAT-3’  F：5’- ACCGAAGTCATAGCCACAC-3’  R：5’- TCAGGAACAGCCACCAG-3’  F：5’- TTCATGTATTGTGTGGGTCTG-3’  R：5’- GGAATCTTGTATTGCATCTGG-3’  F：5’- GCGTCTCCTGAACCTGAGTA -3’  R：5’- CCCTGCTTGTACAGCTCCAG-3’  F：5’- AGCCCTCATCGACAGAAACA-3’  R：5’- GAGAAGTGGCATGCATGAGG-3’  F：5’- GGTTGTCTCCTGCGACTTCA-3’  R：5’- TGGTCCAGGGTTTCTTACTC-3’  F：5’- TCCGGAGAGGAGACTTCACA-3’  R：5’- GTGACTCCAGCTTATCTCTTGGT-3’  F：5’- TGCCACCTTTTGACAGTGATG-3’  R：5’- AAGGTCCACGGGAAAGACAC-3’  F：5’- ACCCTCACACTCACAAACCA-3’  R：5’- ATAGCAAATCGGCTGACGGT-3’ |

**Supplemental diagram**

**Figure S1: Chemical structure and anti-influenza viral effect of lapatinib**

A: Chemical structure of lapatinib; B: Anti-influenza viral effects of lapatinib, A549 cells infected with H1N1 were treated with different concentrations of lapatinib, and the expression of viral genes was detected after 48 hours. Treatments included: lapatinib: lap-6.25 (6.25 μM), lap-3.125 (3.125 μM) and lap-1.56 (1.56 μM). Data are presented as mean ± SD. *P < 0.05, **P < 0.01, ***P < 0.0001.

**Figure S2: Differential gene GO enrichment analysis**

A, B & C shows the GO enrichment analysis of up-regulated and down-regulated differential genes for RSV vs Mock, Lapatinib vs RSV and Lapatinib vs Mock, respectively, with up-regulated genes in red and down-regulated genes in blue.

**Figure S3: KEGG enrichment analysis of differentially expressed genes**

A: RSV vs Mock significantly down-regulated gene pathway enrichment analysis; B: Lapatinib vs RSV significantly up-regulated gene pathway enrichment analysis; C, D: Lapatinib vs Mock significantly up- and down-regulated gene pathway enrichment analysis.

**Figure S4: IL-17 overexpression validation**

A: The expression of IL-17 gene in each group was detected by RT‒qPCR, in which Conrol used culture medium as control and Mock group used empty plasmid as control; B: Protein level detection of overexpression IL-17 gene; C: Analyze the grayscale value of the B by image J. Data represent mean ± SD. *P < 0.05, **P < 0.01, ***P < 0.001.
